# Quantitative trait loci of stripe rust resistance in wheat

**DOI:** 10.1007/s00122-013-2159-9

**Published:** 2013-08-17

**Authors:** G. M. Rosewarne, S. A. Herrera-Foessel, R. P. Singh, J. Huerta-Espino, C. X. Lan, Z. H. He

**Affiliations:** 1 Crop Research Institute, Key Laboratory of Biology and Genetic Breeding in Wheat (Southwest), Sichuan Academy of Agricultural Science, #4 Shizishan Rd, Jinjiang, 610066 Chengdu, Sichuan Province People’s Republic of China; 2 International Maize and Wheat Improvement Centre, (CIMMYT) Apdo., Postal 6-6-41, 06600 Mexico, DF, Mexico; 3 Campo Experimental Valle de Mexico-INIFAP, Apartado Postal 10, 56230 Chapingo, Edo. de Mexico Mexico; 4 Crop Science Institute, Chinese Academy of Agricultural Sciences, 12 Zhongguancun South St, 100081 Beijing, China

## Abstract

*****Key message***:**

**Over 140 QTLs for resistance to stripe rust in wheat have been published and through mapping flanking markers on consensus maps, 49 chromosomal regions are identified.**

**Abstract:**

Over thirty publications during the last 10 years have identified more than 140 QTLs for stripe rust resistance in wheat. It is likely that many of these QTLs are identical genes that have been spread through plant breeding into diverse backgrounds through phenotypic selection under stripe rust epidemics. Allelism testing can be used to differentiate genes in similar locations but in different genetic backgrounds; however, this is problematic for QTL studies where multiple loci segregate from any one parent. This review utilizes consensus maps to illustrate important genomic regions that have had effects against stripe rust in wheat, and although this methodology cannot distinguish alleles from closely linked genes, it does highlight the extent of genetic diversity for this trait and identifies the most valuable loci and the parents possessing them for utilization in breeding programs. With the advent of cheaper, high throughput genotyping technologies, it is envisioned that there will be many more publications in the near future describing ever more QTLs. This review sets the scene for the coming influx of data and will quickly enable researchers to identify new loci in their given populations.

## Stripe rust resistance in wheat

Stripe (or yellow) rust, caused by *Puccinia striiformis* Westend. f. sp. t*ritici* Erikss., is an important biotic constraint of wheat production globally, with regular epidemics occurring in almost all areas where wheat is grown. Control of this disease can be achieved through the timely use of fungicides although this can be expensive to resource poor farmers, and ineffective if not completed in a timely fashion. Furthermore, unprotected neighboring crops that are heavily infected act as reservoirs of inoculums, continually bombarding fungicide treated crops that can ultimately succumb to the disease. Genetic resistance is a more effective way to control the disease as once the resistant variety is sown; no further effort is required by the farmer in relation to disease control.

Resistance genes to fungal diseases in plants can be broadly categorized into two main classes, namely, major and minor resistance genes. Major genes were used by Flor (1956) to describe the complementary gene-for-gene interaction between flax and flax rust. This work highlights the race-specificity of these major genes and implies the non-durable nature of such resistance mechanisms. Generally these genes are involved in a host response to the invading pathogen very early in the infection process and elicit hypersensitivity in which plant host cells that are in close proximity to the invading fungus, or are being attacked by the fungus, undergo programmed cell death. This stops the fungus from establishing feeding structures within the plant, ultimately leading to fungal death. Effective major resistance genes eliminate, or significantly reduce, the ability of the fungal pathogen to reproduce, placing a strong selection pressure in the fungus to evolve and overcome the resistance gene. History shows that when a single major gene protects a large area of a wheat growing region, the fungus can overcome this resistance in a relatively short period of time. Some advanced lines even lose their resistance before or just after release. These types of genes have also been termed seedling genes as they are effective in both seedlings and adult plants; however, a more accurate description would be all-stage resistance (Lin and Chen 2007).

Minor resistance genes generally have a different mode of action and do not provide the immunity, or high level of resistance, that a single major gene does. These genes have variously been called horizontal, partial, non-race specific, slow-rusting, durable or adult plant resistances (Caldwell 1968: Johnson 1988; Parlevliet 1975; Van der Plank 1963). The mechanisms by which fungal disease is inhibited by minor resistances include an increase in the latency period, reduced uredinia size, reduced infection frequency and reduced spore production (Caldwell 1968; Ohm and Shaner 1976; Parlevliet 1975). The additive nature of these genes has long been known with transgressive segregation of resistance in progeny of certain crosses being observed by Farrer (1898). More recently, Singh et al. (2000a) developed wheat lines with near-immunity to stripe rust based on four to five minor resistance loci.

With the advent of modern molecular mapping techniques, our understanding of the location and numbers of adult plant resistance loci has steadily increased. Quantitative trait mapping in wheat was first applied to stripe rust at the turn of the 21st century (Börner et al. 2000; Singh et al. 2000b) and since then there has been over 30 publications describing QTL mapping for stripe rust resistance. As each paper generally describes multiple QTLs, there have been over 140 loci described. There is a large amount of redundancy in these loci as many of the more useful ones are common amongst the different studies.

**Table 1:** Summary of stripe rust regions on group 1 chromosomes associated with stripe rust resistance QTLs

Chromosome region	Source	Markers	Field			Infection type			References
			LOD	PEV	Freq	LOD	PEV	Freq	
QRYr1A.1	Janz	*Xgwm164*	3.0–3.3	6.5–7.0	4/6				Bariana et al. (2010)
QRYr1A.1	Renan	*Xfba118b*	6.5	9.4	½				Dedryver et al. (2009)
QRYr1A.1	Pastor	*wPt-6005* *(Xgwm497)*	4.9	3.6–4.1	2/4				Rosewarne et al. (2012)
QRYr1A.2	Naxos	*Xwmc59* *Xbarc213*	3.8	8.2	1/3				Ren et al. (2012a)
QRYr1B.1	Pastor	*wPt-6240* *(Xgwm11, Xgwm273)*	6.6	5.0–5.1	2/3				Rosewarne et al. (2012)
QRYr1B.2	Express	*Xwmc631* *Xgwm268*	3.1–4.1	4.5–5.2	2/3			0/3^b^	Lin and Chen (2009)
QRYr1B.3	Kukri	*Xbarc80*	3.1	6	2/6				Bariana et al. (2010)
QRYr1B.3	Brigadier	*Xwmc735*	3.3–4.5	7.9–13.1	2/2	3.2	9.9	½^b^	Jagger et al. (2011)
QRYr1B.3	Guardian	*Xgwm259* *Xgwm818*	5.7–11.7	15–45	2/2	3.8–6.5	10–22	2/2^b^	Melichar et al. (2008)
QRYr1B.3	Pavon 76	*Xgwm259*	11–17	48–56	3/3	6.3	18.5	1/1^b^	William et al. (2006)
QRYr1B.3	CPI133872	*wPt-1313* *(Xwmc44, Xgwm259, Xgwm140)*	2.8–4.8	10–17	¾				Zwart et al. (2010)
QRYr1B.3	CD87	*Xpsr305*	14.3^a^	9	2/2				Bariana et al. (2001)
QRYr1B.3	Saar	*Xwmc719* *Xhbe248*	3.8	17.4	1/1				Lillemo et al. (2008)
QRYr1B.3	Attila	*LTN2*	13–24	33–64	3/3				Rosewarne et al. (2008)
QRYr1B.3	Pastor	*XcsLV46*	23	15.1–22.1	3/3				Rosewarne et al. (2012)
QRYr1D.1	CPI133872	*Xwmc147*	3	13	¼				Zwart et al. (2010)
QRYr1D.1	Stephens	*379227* *Xbcd1434*	3.3	11	½				Vazquez et al. (2012)
QRYr1D.2	Naxos	*Xwmc432*	3.9	5.8	1/3				Ren et al. (2012a)

Markers in parentheses were not described in the original publication but were used as closely associated substitutes on consensus maps where the original markers were not available. Frequency (Freq) describes to number of environments that the locus had a significant QTL, out of the total number of environments tested

^a^Likelihood ratio reported

^b^Infection type scored in field on adult plants

## Marker technologies

Concurrent to the detailed rust QTL work, marker systems have also developed rapidly, with the earlier maps described with RFLP markers. This evolved to include the much easier to apply SSR markers with their genetic location being well documented through many different mapping studies. Other PCR-based marker systems have been implemented and include RGAP, CAPS, EST, STS, AFLP and RAPD markers. More recently, multiplex platforms of SNP, DArT and whole genome sequencing markers make the production of detailed maps commonplace. A major issue with all of these marker systems is that they are not yet well integrated. SSR markers, being the best categorized, are often used to link maps that contain different marker types and build consensus between different maps. Although this has limitations, mainly around the micro-order of specific markers between maps, it does help to locate useful traits within the genome as well as describing chromosomal reordering through translocations.

## Consensus mapping

The incorporation of quantitative resistances to facilitate near-immunity is seen as providing durable resistance and has been the breeding strategy in the CIMMYT wheat breeding program since the 1960s. Breeding for durability is becoming a priority in many breeding program globally as they move away from major gene resistances. It is therefore critical to gain a better understanding of the location and breeding values of these loci. In this review, all known stripe rust QTLs are located on consensus maps by using the published information on flanking markers in an effort to locate their position. Consensus mapping of flanking markers has the ability to differentiate important regions on individual chromosomes as well as highlighting important loci that have been identified in many studies. It does have the limitation that loci falling in the same chromosomal region cannot be differentiated; however, it does identify the minimum number of regions contributing to resistance and gives insights into future studies that could lead to gene discoveries.

The consensus maps used were located on the cmap website (http://ccg.murdoch.edu.au/cmap/ccg-live/) with the main maps being “Consensus Map August 11 2003” “Somers Consensus April 04” and “Consensus 2010-11”, the latter being a series of single chromosome consensus maps developed over the years 2010 and 2011. Consensus 2010-11 maps generally had the best coverage of markers and contained the most markers. These consensus maps were used as the basis for all figures, although on occasion, map positions of some markers were inferred from the earlier mentioned consensus maps. Some of the DArT markers were not in any of the consensus maps, and their position was also inferred from flanking SSR markers in other mapping populations. The identification of specific chromosomal regions were determined by either by a position of a single reported QTL, or more commonly by the clustering of flanking markers from two or more studies. Often, the flanking markers from different studies identified overlapping segments and the limits of each region were set by the outermost flanking markers from these segments. Regions were labeled according to the following nomenclature: QRYrChromosome.position, where QR stands for QTL Region, Yr for stripe (yellow) rust, Chromosome conventional name (group number 1–7 followed by genome A, B or D) and position, where regions were labeled numerically with the first position being the region closest to the telomere of the short arm of that respective chromosome.

QTL studies have used different software to describe aspects of QTLs, of particular note is the variance explained by the relevant QTL. Some packages have used *R* ^2^ to describe this term but for consistency we use the term Phenotypic Explained Variance (PEV) throughout the text.

## QTL regions associated with stripe rust resistance

There were 47 regions identified that had an effect against stripe rust severity and these were found on all chromosomes with the exception of 5D. Many of the regions are known to contain more than one gene but limitations with consensus mapping did not permit further delineation of this region. For example, chromosome 2B had a region that contained *Yr27* and *Yr31*, two linked race-specific seedling genes, as well as a number of QTLs that were effective in the adult plant stage. This suggests a minimum number of regions have been identified. Described below are seven sections relating to each of the different chromosome groups of wheat, sequentially highlighting regions where stripe rust QTLs have been found and indicating which regions are most important.

## Group 1

**Fig. 1:**
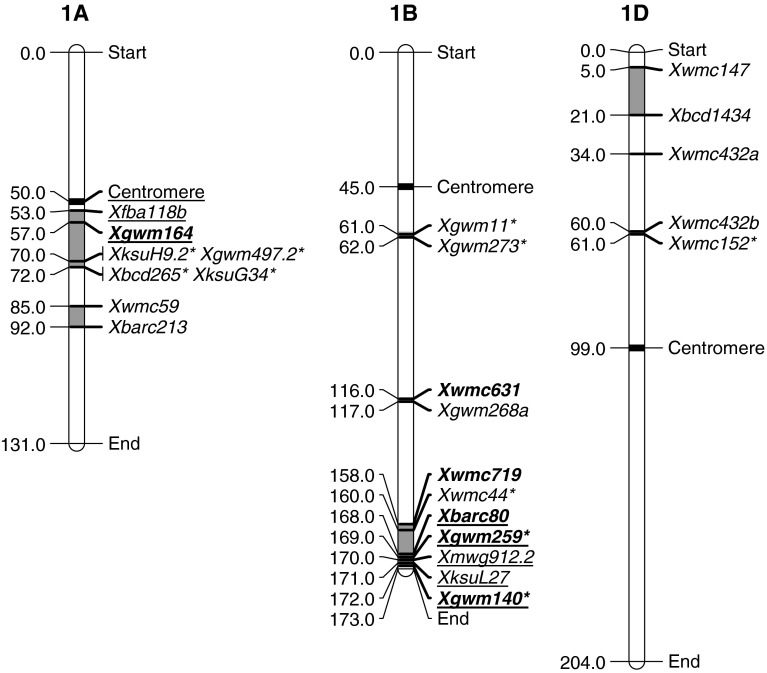
Location of flanking markers associated with stripe rust QTLs on group 1 chromosomes. Marker locations are taken from consensus maps from the CMap website (http://ccg.murdoch.edu.au/cmap/ccg-live/). Map distances for markers in plain text are from “Consensus Maps 2010-11”, underlined markers have map positions estimated from “Consensus 2003” and markers in bold have map positions estimated from “Sommers 2004” consensus maps. *Identified markers that were used as associated (linked) substitutes for QTL markers that were not available on any consensus maps

The QTLs associated with group 1 chromosomes are listed in Table 1 and shown diagrammatically in Fig. 1. Chromosome 1A had four QTLs described (Bariana et al. 2010; Dedryver et al. 2009; Ren et al. 2012a; Rosewarne et al. 2012). The flanking markers of these form two distinct groups although they are both on the long arm of chromosome 1A. The QTLs from Janz, Renan and Pastor are proximal on 1AL, while flanking markers from Naxos appear to be somewhat more distal on 1AL. All four QTLs resulted in similar LOD and PEV scores (Table 1), but they are relatively inconsistent in that they are picked up in approximately half of the environments tested. Given the closeness of the Naxos QTL with the other three, there is a possibility that they are at the same locus. The PEV scores are moderate and this is a locus of intermediate value. Application of markers across the populations would help to clarify if there are two regions of importance for stripe rust on this chromosome.

Chromosome 1B contains the important locus of *Lr46/Yr29*, hereafter referred to as *Yr29.* This had been identified in numerous studies and is described as group 3 on 1BL (QRYr1B.3) in Table 1 (Bariana et al. 2001, 2010; Jagger et al. 2011; Lillemo et al. 2008; Melichar et al. 2008; Rosewarne et al. 2008, 2012; William et al. 2006; Zwart et al. 2010). There is a wide range in the LOD (2.8–23) and PEV (4.5–65) scores indicating the variability that this locus can contribute. However, *Yr29* is important as is demonstrated by the number of significant environments tested. Nearly every study finds this locus significant in every environment tested with the exception of Bariana et al. (2010). Interestingly, Bariana et al. (2010) also had *Lr34/Yr18* (hereafter termed *Yr18*) segregating in that population and it is now becoming apparent that when those two loci are in the same genetic background, the *Yr29* locus has lesser effect (Lillemo et al. 2008; Suenaga et al. 2003; Yang et al. 2013). This is clearly a useful locus on which to build a basis for multi-genic resistance. Chromosome 1B also had two other regions that contributed to stripe rust resistance. These are both also likely to be on 1BL with the proximal locus identified by Rosewarne et al. (2012) in Pastor. This was effective in two of three environments tested and had intermediate LOD and PEV scores. A QTL identified by Lin and Chen (2009) from the cultivar Express was clearly located between the proximal Pastor QTL and the *Yr29* locus. In their paper, they could not conclude whether their locus was different from *Yr29* although clearly it falls in a different position in the consensus maps and is likely to be a different gene.

There were three QTLs identified on Chromosome 1DS (Ren et al. 2012a; Vazquez et al. 2012, Zwart et al. 2010), all with relatively minor and inconsistent effects against the pathogen. These loci probably form two separate regions with the Stephens (Vazquez et al. 2012) and CPI133872 (Zwart et al. 2010) QTLs being in the telomeric region of 1DS. The location of the Naxos locus was ill-defined as *Xwmc432* has two forms in the consensus maps that are 26 cM apart and Ren et al. (2012a) did not differentiate which form was associated with the Naxos resistance. However, it would appear that *Xwmc432b* was the allele identified, as this marker was mapped close to *Xwmc152*, a marker associated with their flanking DArT marker *wPt*-*6979*. Either way, the identification of two minor loci on chromosome 1DS is still supported.

## Group 2

**Fig. 2:**
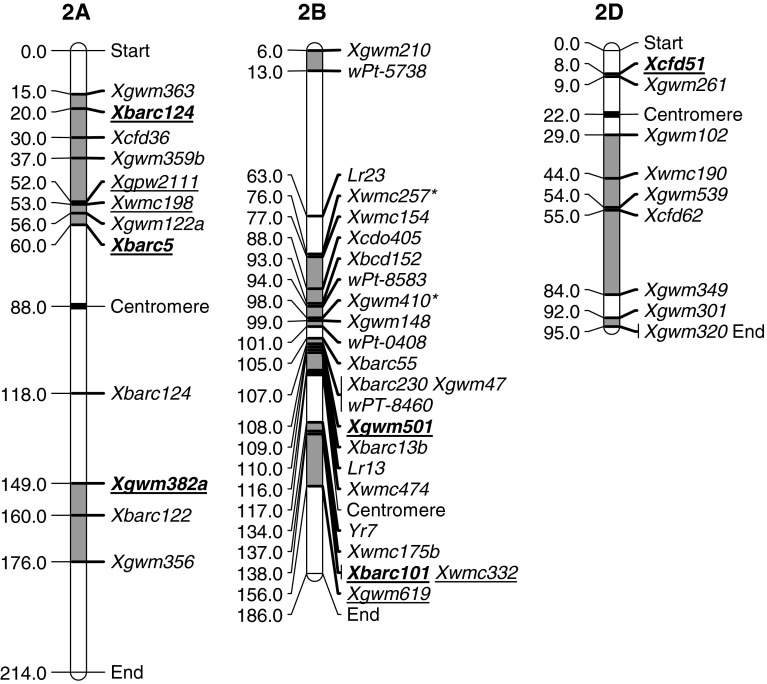
Location of flanking markers associated with stripe rust QTLs on group 2 chromosomes. Marker locations are taken from consensus maps from the CMap website (http://ccg.murdoch.edu.au/cmap/ccg-live/). Map distances for markers in plain text are from “Consensus Maps 2010-11”, underlined markers have map positions estimated from “Consensus 2003” and markers in bold have map positions estimated from “Sommers 2004” consensus maps. *Identified markers that were used as associated (linked) substitutes for QTL markers that were not available on any consensus maps

The group 2 chromosomes have several important regions for stripe rust resistance and these are outlined in Table 2 and Fig. 2. Chromosome 2A has one region associated with resistance on the short arm and another region on the long arm. Both Récital (Dedryver et al. 2009) and Camp Remy (Boukhatem et al. 2002; Mallard et al. 2005) have QTLs in both of these regions. The region around the 2AS QTLs are also associated with the major, race-specific gene *Yr17*, introgressed from *Aegilops ventricosa* (Bariana and McIntosh 1993). Both Pioneer 26R61 (Hao et al. 2011) and Y16DH70 (Agenbag et al. 2012) have QTLs with LOD scores consistent with major genes in this region although markers confirmed that the alien introgression containing *Yr17* was not present. It seems likely that these lines contain potentially new major genes for resistance. The other QTLs in the 2AS region appeared to be more consistent with minor resistance genes where the LOD scores were lower and effect across environments was generally inconsistent. Both the 2AS and 2AL minor resistance genes are likely to be useful in contributing to stable resistance.

**Table 2:** Summary of stripe rust regions on group 2 chromosomes associated with stripe rust resistance QTLs

Chromosome region	Source	Markers	Field			Infection type			References
			LOD	PEV	Freq	LOD	PEV	Freq	
QRYr2A.1	Y16DH70	*Xgwm636*	14–19	37–49	2/2	9–16	31–53	2/2^b^	Agenbag et al. (2012)
QRYr2A.1	Pioneer26R61	*Xbarc124* *Xgwm359*	15–25	23–24	3/3				Hao et al. (2011)
QRYr2A.1	Recital	*Xcfd36*	5.0–6.8	5.5–7.7	2/4				Dedryver et al. (2009)
QRYr2A.1	Camp Remy	*Xgwm356* *Xgwm122, Xgpw2111*	5.4–6.1	20–40	4/4				Mallard et al. (2005)
QRYr2A.1	Stephens	*wPt-0003* *(Xgwm359)*	3.8–6.7	9–20	4/6				Vazquez et al. (2012)
QRYr2A.1	Kukri	*Xbarc5*	3.7–4.1	13–15	2/6				Bariana et al. (2010)
QRYr2A.1	*T*. *monococcum*	*Xwmc170* *(Xbarc5)*	7.8–15	7–12	3/3	13–15	11–13	3/3^b^	Chhuneja et al. (2008)
QRYr2A.2	Recital	*Xgwm382* *Xbarc122*	4.4–5.4	4.5–8.1	2/4				Dedryver et al. (2009)
QRYr2A.2	Camp Remy	*Xgwm359* *Xgwm382*	1.7	10.7	2/2	3	15	2/2^b^	Boukhatem et al. (2002)
QRYr2B.1	Renan	*Xgwm210a* *Xfbb67c*	12–13	9–16	4/4				Dedryver et al. (2009)
QRYr2B.1	Stephens	*wPt-5738*	3–3.6	10	2/6				Vazquez et al. (2012)
QRYr2B.2	Attila	*Xwmc257* *Xwmc154*		4.7–9.3	3/3				Rosewarne et al. (2008)
QRYr2B.2	Luke	*Xwmc154* *Xgwm148*				5.1–7.7	32–37	3/3^b^	Guo et al. (2008)
QRYr2B.2	Opata 85	*Xcdo405, Xbcd152*				7.4	30.7	2/2^b^	Boukhatem et al. (2002)
QRYr2B.2	Chapio	*wPt-0079* *(wPt-8583, Xgwm410)*	3.3–7.8	4.9–13.6	3/8				Yang et al. (2013)
QRYr2B.2	Luke	*Xgwm148* *Xbarc167*				5.5–8.8	33–42	3/3^b^	Guo et al. (2008)
QRYr2B.2	Kareiga	*Xgwm148*	55^a^	30	1/1	95^a^	46	1/1^b^	Ramburan et al. (2004)
QRYr2B.2	Stephens	*wPt-0408*	2.8–4.3	8–13	5/6				Vazquez et al. (2012)
QRYr2B.3	Pingyuan 50	*Xbarc13* *Xbarc55*	1.7–3.2	5.1–9.5	¾				Lan et al. (2010)
QRYr2B.3	Louise	*Xwmc474* *Xbarc230*	5.5–30	11–58	7/8				Carter et al. (2009)
QRYr2B.3	Camp Remy	*Xgwm47* *Xgwm501*	11.8	46	2/2	12	46	2/2^b^	Boukhatem et al. (2002)
QRYr2B.3	Naxos	*wPt-8460*	4.9	12.2	1/3				Ren et al. (2012a)
QRYr2B.3	Pastor	*Yr31* *(Lr23, Lr13)*	10–17	32–66	¾				Rosewarne et al. (2012)
QRYr2B.4	Cranbrook	*Xwmc339* *Yr7*							Bariana et al. (2001)
QRYr2B.4	Camp Remy	*Xbarc101* *Xgwm120, Xwmc175*	22–36	42–61	4/4				Mallard et al. (2005)
QRYr2B.4	Aquileja	*Xwmc175* *Xwmc332*				7.8–13.9	49–62	3/3^b,c^	Guo et al. (2008)
QRYr2B.4	Avocet	*Xgwm619*	3.6	6.3	1/3				Rosewarne et al. (2008)
QRYr2D.1	Libellula	*Xcfd51* *Xgwm261*	5–10	8–10	2/4				Lu et al. (2009)
QRYr2D.2	Camp Remy	*Xgwm102* *Xgwm539*	5.7–11	24–69	4/4				Mallard et al. (2005)
QRYr2D.2	Yr16DH70	*Xgwm102*	5.4	8.4	1/2	6.1–6.6	10	2/2^b^	Agenbag et al. (2012)
QRYr2D.2	Sunco	*Xgdm005* *Xwmc190*	15.8^a^		1/1				Bariana et al. (2001)
QRYr2D.2	Guardian	*Xgwm539* *Xgwm349*	4.4	11	½	4.3	14	½^b^	Melichar et al. (2008)
QRYr2D.2	Naxos	*Xgwm539* *Xcfd62*	5.6	10.2	1/3				Ren et al. (2012a)
QRYr2D.2	Fukuho-komugi	*Xgwm349*	14.5^a^	9.6	1/3				Suenaga et al. (2003)
QRYr2D.3	Alcedo	*Xgwm320* *Xgwm301*	14–18	32–36	2/2	18–28	36–53	2/2^b^	Jagger et al. (2011)

Markers in parentheses were not described in the original publication but were used as closely associated substitutes on consensus maps where the original markers were not available. Frequency (Freq) describes to number of environments that the locus had a significant QTL, out of the total number of environments tested

^a^Likelihood ratio reported

^b^Infection type scored in field on adult plants

^c^Stripe number per 10 cm^2^ leaf area

There are at least four regions associated with rust resistance on chromosome 2B. The QRYr2B.1 region was identified by Dedryver et al. (2009) and Vazquez et al. (2012) with the marker *Xgwm210* and *wPt*-*5738* respectively, placing this region at the telomere of 2BS.

The QRYr2B.2 region was identified in six studies and coincides with the location of several seedling resistance genes. Rosewarne et al. (2008) indicated that Attila had *Yr27* and Yang et al. (2013) identified *Yr31* in Chapio, both falling in this region. Pastor (Rosewarne et al. 2012) also contained *Yr31*, with this gene being flanked by the leaf rust seedling resistance genes *Lr23* and *Lr13*. The cultivar Luke (Guo et al. 2008) contained two loci, 23 cM apart, in this region that were observed with infection type data and were shown to be high temperature adult plant (HTAP) resistances. Opata 85 (Boukhatem et al. 2002) also contained a major QTL identified through IT data in this region and Ramburan et al. (2004) used IT, final disease severity and seedling data to show an adult plant resistance with major effect in Kariega. Finally, Vazquez et al. (2012) used final disease severity to identify a highly significant QTL in the majority of environments tested. The combination of these data show that QRYr2B.2 is a gene rich region which contains a number of seedling and HTAP resistance genes, as shown by relatively high LOD and PEV scores. Furthermore, these types of genes were effective across most environments, with only *Yr31* not being significant in environments where a virulent pathotype was used (Rosewarne et al. 2012; Yang et al. 2013).

The next region also contained a seedling resistance gene derived from Camp Remy (Boukhatem et al. 2002), HTAP resistance in Louise (Carter et al. 2009) and minor QTLs in Pingyuan50 (Lan et al. 2010) and Naxos (Ren et al. 2012a). This region was located close to QRYr2B.2 and although the flanking markers from the loci within these two regions did not overlap, limitations in consensus mapping do not rule out the possibility that the same genes may contribute to some resistances in both regions. Although further studies are required to separate individual genes, it is clear that the QRYr2B.2 and QRYr2B.3 regions have been used extensively in resistance breeding.

The fourth region on 2BS was also associated with seedling resistances with Bariana et al. (2001) mapping *Yr7* from Cranbrook. Mallard et al. (2005) asserted that the 2BL QTL from Thatcher was most likely this same gene. Our study supports this as the Mallard et al. (2005) markers fall very closely to *Yr7* in the consensus maps. A locus from Aquileja (Guo et al. 2008) also clusters in this region and showed seedling resistance. The high LOD and PEV scores and its effectiveness in all three environments support this being a major gene; however, it is not *Yr7* as the pathotype used (CYR32) is virulent on *Yr7*. Mallard et al. (2005) point out that *Yr7* and *Yr5* are probably allelic so it seems the Aquileja 2BL locus is likely to contain the latter, with CYR32 being avirulent on this gene. The final QTL in this region was identified in Avocet-*YrA* (hereafter termed Avocet) (Rosewarne et al. 2008), a line often used as a susceptible parent. Other minor QTLs from Avocet have been identified on 3A, 4B, 6A and 7A (Lillemo et al. 2008; Rosewarne et al. 2012; William et al. 2006). These loci invariably have relatively low LOD and PEV scores and are often inconsistent across environments. These very minor QTLs still provide some level of resistance as transgressive segregation can sometimes be observed in Avocet crosses where a progeny line is more susceptible than Avocet (Melichar et al. 2008; Rosewarne et al. 2008, 2012).

There are three regions associated with resistance on chromosome 2D. One region on 2DS was identified in a single study with Libellula (Lu et al. 2009). This locus had intermediate LOD and PEV scores and was significant in two of the four environments tested. This characteristic minor gene can contribute to durable resistance when combined with a number of other loci.

There was a large region in the proximal area of 2DL that contained QTLs from 6 parents. The flanking markers for the Guardian QTL were *Xgwm539* and *Xgwm349*. There is conjecture in the linkage maps as to how close these markers are, with Somers Grain Genes Consensus having them 2.3 cM apart, yet Consensus 2003 and Consensus 2010 maps had them about 30 cM apart. QTLs from Camp Remy (Mallard et al. 2005) and Naxos (Ren et al. 2012a) also had *Xgwm539* as a flanking marker and Fukuho-komugi had *Xgwm349* as a flanking marker. Flanking markers from YrDH70 (Agenbag et al. 2012) and Sunco (Bariana et al. 2001) also fall within this region as does the adult plant resistance gene *Yr16* (Worland and Law 1986). The loci outlined here had inconsistent effects across environments as has been observed with other minor loci. Further work would be required to differentiate all of these loci from *Yr16*; however, it still appears to be a valuable locus in combination with other genes.

A final locus, QRYr2D.3 was identified close to the telomere of 2DL in Alcedo and was effective in both seedling and adult plant stages. The high LOD and PEV scores support a major gene for resistance in this region.

## Group 3

**Fig. 3:**
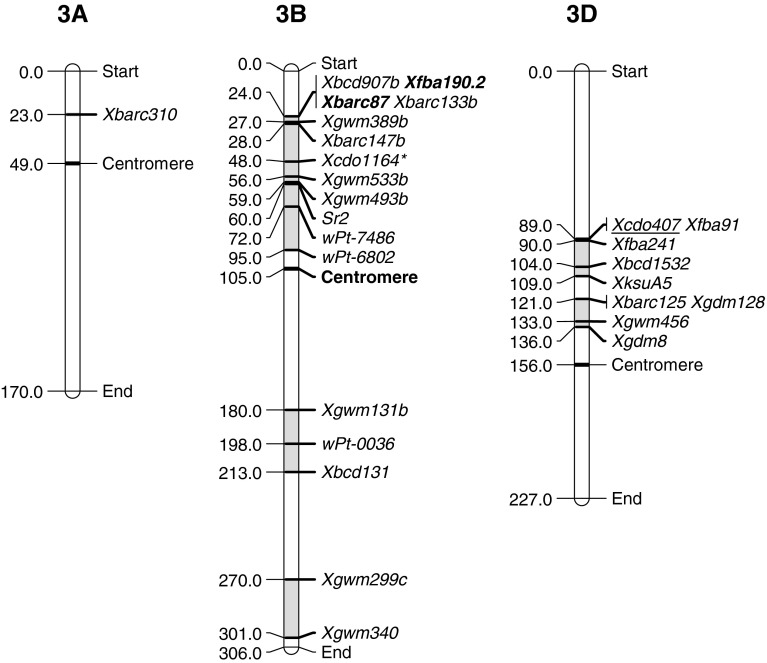
Location of flanking markers associated with stripe rust QTLs on group 3 chromosomes. Marker locations are taken from consensus maps from the CMap website (http://ccg.murdoch.edu.au/cmap/ccg-live/). Map distances for markers in plain text are from “Consensus Maps 2010-11”, underlined markers have map positions estimated from “Consensus 2003” and markers in bold have map positions estimated from “Sommers 2004” consensus maps. *Identified markers that were used as associated (linked) substitutes for QTL markers that were not available on any consensus maps

There were 14 studies that identified QTLs on the group 3 chromosomes. Lillemo et al. (2008) identified a 3AS QTL in Saar but this was only investigated in one stripe rust environment so its breeding value is yet to be determined. The 3B chromosome appears to have at least three regions associated with stripe rust resistance. Consensus mapping in this region was difficult as a group of SSR markers are multi-allelic and produced up to three bands each that were located in different positions of the chromosome. Most of the publications do not discriminate which band was identified. For example, *Xbcd907*, *Xgwm389*, *Xbarc147*, *Xbarc133*, *Xgwm533* and *Xgwm493* have at least an “a” and a “b” locus, with the “a” loci of these markers clustering near the telomere of 3BS, whereas the “b” loci cluster more towards the middle of 3BS. We have presented data based on the “b” clustering loci; however, we cannot be sure if some reported QTLs that make up this cluster are from the “a” locus region. Nonetheless, the majority of QTLs identified on 3B are on the short arm. The consensus mapping approach can only give a broad picture of chromosomal regions that are involved in resistance. For example, Hao et al. (2011) identified three QTLs that all fall within QRYr3B.1, suggesting that there is more than one locus contributing to resistance in this region. However, QTL analyses often report multiple peaks within a region that can be brought together by reordering the markers. The QRYr3B.1 region is known to be extremely important as it is the location of *Yr30*, a partial resistance gene that is very tightly linked, or pleiotropic, to the slow-stem rusting locus *Sr2* (shown in Fig. 3) and the phenotypic marker of pseudo-black chaff (*Pbc*). *Yr30* is a valuable gene that has shown to work well in combination with other genes such as *Yr18* (Yang et al. 2013) and gives an intermediate effect in most environments. It is expressed only in the adult plant stage as William et al. (2006) did not find any evidence of resistance at this locus in a seedling assay. Singh et al. (2000b) and William et al. (2006) did find a significant QTL at this site with IT data on adult plants, and this chlorotic effect can be seen in advanced stripe rust infections on lines protected solely by adult plant resistances (Singh et al. 2005). The LOD and PEV scores within the studies listed in Table 3 indicate that QRYr3B.1 has a consistent intermediate effect on stripe rust and is fairly consistent across environments. Bariana et al. (2010) described a QTL from Kukri on 3BS and assumed it was *Yr30*. We have included the main DArT marker *wPt*-*6802* used by Bariana et al. (2010) in consensus mapping and this extends Group QRYr3B.1 by 23 cM. However, it should be noted that the Bariana et al. (2010) study describes a significant region of over 82 cM, and this region also includes *Xgwm533.* This SSR marker has been associated with *Yr30* in four other studies (Börner et al. 2000; Dedryver et al. 2009; Spielmeyer et al. 2005; William et al. 2006).

**Table 3:** Summary of stripe rust regions on group 3 chromosomes associated with stripe rust resistance QTLs

Chromosome region	Source	Markers	Field			Infection type			References
			LOD	PEV	Freq	LOD	PEV	Freq	
QRYr3A.1	Saar	*Xstm844tcac* *Xbarc310*		8.7	1/1				Lillemo et al. (2008)
QRYr3B.1	AGS2000	*wPt-2557* *Xbarc133*	3.8	5	1/3				Hao et al. (2011)
QRYr3B.1	Chapio	*Xbarc147* *wPt-3038*	3.8–18	4.9–15	7/8				Yang et al. (2013)
QRYr3B.1	Oligoculm	*Xgwm389*	23.1^a^	2.9–4.9	¾				Suenaga et al. (2003)
QRYr3B.1	Opata 85	*Xfba190* *Xbcd907*	3.9	16	1/3	7.2	28	1/1^b^	Singh et al. (2000b)
QRYr3B.1	AGS2000	*wPt-730063* *wPt-9579*	3.6	7	1/3				Hao et al. (2011)
QRYr3B.1	AGS2000	*wPt-1612* *wPt-7486*	3.1–3.2	4–5	2/3				Hao et al. (2011)
QRYr3B.1	Lgst.79-74	*Xgwm533* *Xgwm493*			2/2				Börner et al. (2000)
QRYr3B.1	Pavon 76	*Xgwm533*	2	4.6–6	0/3	2.7	8.3	1/1^b^	William et al. (2006)
QRYr3B.1	Renan	*Xgwm533*	4.1–7.7	3.3–11	2/2				Dedryver et al. (2009)
QRYr3B.1	Kukri	*wPt-6802*	2.6	5	1/6				Bariana et al. (2010)
QRYr3B.2	Pastor	*wPt-2458* *wPt-0036*	16.2	3.8–5.8	¾				Rosewarne et al. (2012)
QRYr3B.2	Renan	*Xgwm121b* *Xbcd131*	4.4	6.3	½				Dedryver et al. (2009)
QRYr3B.3	Express	*Xgwm340* *Xgwm299*	5.2–8.4	9.1–13	3/3	2.6–5	6–11	3/3^b^	Lin and Chen (2009)
QRYr3D.1	Opata 85	*Xcdo407* *XksuA5*				2.8	1	1/1^b^	Boukhatem et al. (2002)
QRYr3D.1	Opata 85	*Xfba241* *Xfba91*	3.5	14	¼				Singh et al. (2000b)
QRYr3D.2	Recital	*Xbarc125* *Xgwm456a*	5.2–6.2	4.7–7.5	2/2				Dedryver et al. (2009)
QRYr3D.2	Chapio	*Xgdm8* *Xgdm128*	3.2–8	3.2–11	3/8				Yang et al. (2013)

Markers in parentheses were not described in the original publication but were used as closely associated substitutes on consensus maps where the original markers were not available. Frequency (Freq) describes to number of environments that the locus had a significant QTL, out of the total number of environments tested

^a^Likelihood ratio reported

^b^Infection type scored in field on adult plants

The second region associated with stripe rust resistance on chromosome 3B was centrally located on the long arm. Renan (Dedryver et al. 2009) and Pastor (Rosewarne et al. 2012) contributed with intermediate to small effect QTLs and these were not consistently detected across environments. The third region associated with resistance was a HTAP gene (Lin and Chen 2009) located near the telomere of 3BL. This locus is clearly different from those mentioned above as it was consistently identified across environments, had an intermediate level of effect and contributed significantly to lowering IT in adult plants.

There were two regions associated with resistance on chromosome 3D. Figure 3 shows that the nearest flanking markers from these two groups are only 12 cM apart and the possibility exists that all of the described QTLs are in the same region. Region QRYr3D.1 was identified from Opata 85 by Boukhatem et al. (2002) in a seedling assay on IT and later confirmed by Singh et al. (2000b) in a field-based assessment of adult plants. The QRYr3D.2 region was identified in Recital (Dedryver et al. 2009) and Chapio (Yang et al. 2013). The intermediate LOD and PEV scores were of the same order as those observed by Singh et al. (2000b) and finer mapping would be required to clearly differentiate QRYr3D.1 and QRYr3D.2.

## Group 4

**Fig. 4:**
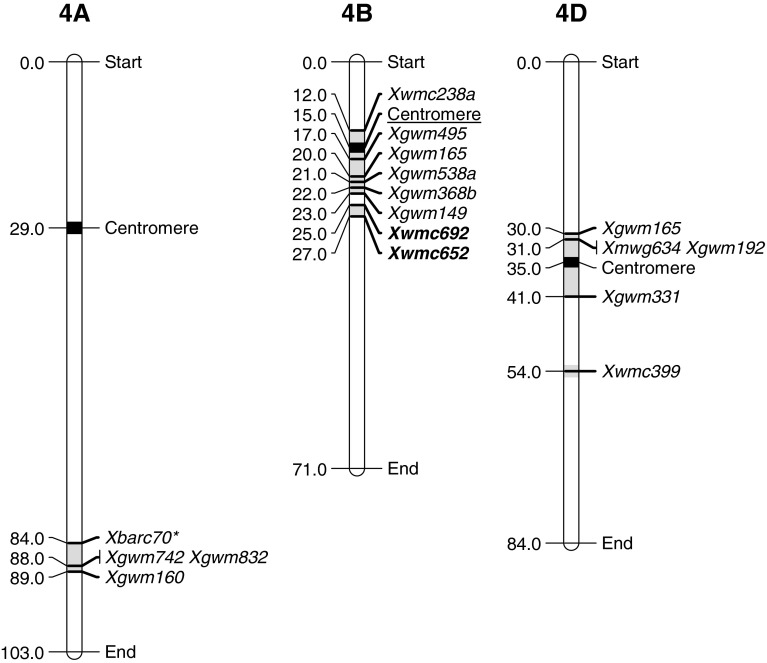
Location of flanking markers associated with stripe rust QTLs on group 4 chromosomes. Marker locations are taken from consensus maps from the CMap website (http://ccg.murdoch.edu.au/cmap/ccg-live/). Map distances for markers in plain text are from “Consensus Maps 2010-11”, underlined markers have map positions estimated from “Consensus 2003” and markers in bold have map positions estimated from “Sommers 2004” consensus maps. *Identified markers that were used as associated (linked) substitutes for QTL markers that were not available on any consensus maps

The group 4 chromosomes had limited QTLs and these are outlined in Table 4 and Fig. 4. Two studies have identified a region contributing to resistance on the long arm of chromosome 4A. Kariega had a locus that conferred all-stage resistance and is therefore likely a major gene (Ramburan et al. 2004). Vazquez et al. (2012) identified a region that had a small effect in one of the two environments tested. They identified this QTL with the DArT marker *wPt*-*9901*, which was not present in any consensus map; however, it was mapped within 1.6 cM of *Xbarc70* in Arina/NK93604 population (Howes pers. comm.). *Xbarc70* is located very close to the Kariega QTL and we have described it as a single region although it may contain two genes.

**Table 4:** Summary of stripe rust regions on group 4 chromosomes associated with stripe rust resistance QTLs

Chromosome region	Source	Markers	Field			Infection type			References
			LOD	PEV	Freq	LOD	PEV	Freq	
QRYr4A.1	Kariega	*Xgwm160*	23^a^	15	1/1	8^a^	6	1/1^c^	Ramburan et al. (2004)
		*Xgwm742, Xgwm832*				40	24	1/1^b^	
QRYr4A.1	Stephens	*wPt-9901* *(Xbarc70)*	3	7	½				Vazquez et al. (2012)
QRYr4B.1	Palmiet	*Xgwm165* *Xgwm495*			0/2	7.6	12	1/2^c^	Agenbag et al. (2012)
QRYr4B.1	Alcedo	*Xwmc692* *Xstm535*	13–15	24–29	2/2	8–18	22–37	3/3^c^	Jagger et al. (2011)
QRYr4B.1	Oligoculm	*Xgwm538*		4.4–12.3	¾				Suenaga et al. (2003)
QRYr4B.1	Avocet	*Xgwm495* *Xgwm368*	2.6–4.4	8–13	3/3	4.7	13	1/1^c^	William et al. (2006)
QRYr4B.1	Janz	*wPt-8543* *Xwmc238, Xgwm368*	3.8–5.2	9–17	¾				Zwart et al. (2010)
QRYr4B.1	Libellula	*Xgwm165* *Xgwm149*	2.9–3.6	4–5	2/4				Lu et al. (2009)
QRYr4B.1	Strampelli	*Xgwm165, Xgwm149*	3	5	1/5				Lu et al. (2009)
QRYr4B.2	Guardian	*Xwmc652* *Xwmc692*	3	5	½	2.5	7	1/2^c^	Melichar et al. (2008)
QRYr4D.1	Bainong 64	*Xgwm165* *Xwmc331*	3	8	1/3				Ren et al. (2012b)
QRYr4D.1	Pastor	*wPt-6880* *wPt-4572 (Xmwg634)*	11.8	2.8–4.9	2/4				Rosewarne et al. (2012)
QRYr4D.1	W-219	*Xmwg634*	2.3–4.4	9–17	2/4	8.6	31	1/1^c^	Singh et al. (2000b)
QRYr4D.1	RL6077	*Xgwm165* *Xgwm192*							Herrera-Foessel et al. (2011)
QRYr4D.2	Oligoculm	*Xwmc399*	32^a^	3–8	4/4				Suenaga et al. (2003)

Markers in parentheses were not described in the original publication but were used as closely associated substitutes on consensus maps where the original markers were not available. Frequency (Freq) describes to number of environments that the locus had a significant QTL, out of the total number of environments tested

^a^Likelihood ratio reported

^b^Infection type scored at seedling stage in glasshouse

^c^Infection type scored in field on adult plants

Chromosome 4B had two regions associated with stripe rust resistance and these were only separated by 2 cM on the consensus maps. Six lines, Alcedo (Jagger et al. 2011), Avocet (William et al. 2006), Janz (Zwart et al. 2010), Libellula, Strampelli (Lu et al. 2009) and Palmiet (Agenbag et al. 2012) were identified with QTLs on the long arm of chromosome 4B. Alcedo had relatively high LOD and PEV scores, whereas all the others had low to moderate scores. Several of the studies used both disease severity and IT data from field studies and showed that these QTLs were mostly consistent across environments (Agenbag et al. 2012; Jagger et al. 2011; William et al. 2006). Both Avocet and Palmiet were the susceptible parent in the respective crosses and QTLs from the susceptible parent have been discussed previously. The lines Libellula and Strampelli (Lu et al. 2009) both have San Pastore in their pedigree and it seems likely that they share the same gene. This locus was not effective in all environments and although it is in a similar position to the other QRYr4B.1 QTLs, the inconsistent nature of this QTL suggests that it may be a different gene. Melichar et al. (2008) identified a QTL (QRYr4B.2) in Guardian with flanking markers *Xwmc692* and *Xwmc652*. In the consensus map, these were 2 cM and 4 cM distal to the last marker in QRYr4B.1 cluster and could easily have been classified as belonging to the QRYr4B.1 group. If so, its comparatively lower LOD and PEV scores make it more similar to the Libellula and Strampelli QTLs rather that the other QTLs observed in this region.

Relatively few QTLs were identified on chromosome 4D, yet it has turned out to contain what is an important and under-utilized locus. Hiebert et al. (2010) and Herrera-Fossel et al. (2011) characterized *Lr67/Yr46/Sr55/Pm46* (hereafter termed *Yr46*) in this region and have shown that it confers partial resistance to multiple pathogens including leaf rust, stripe rust, stem rust and powdery mildew. Furthermore it also confers the phenotypic marker “leaf tip necrosis” (*Ltn3*), making it very similar to the *Yr18* and *Yr29* loci. These types of loci, when combined with two to three other minor loci, can provide near-immunity to stripe rust. Bainong 64 (Ren et al. 2012b), Pastor (Rosewarne et al. 2012) and W-219 (Singh et al. 2000b) also contained QTLs with intermediate LOD scores in this region and these were effective in approximately half of the environments tested. It is possible that some of these QTLs correspond to *Yr46* with Ren et al. (2012b) reporting both stripe rust and powdery mildew QTL at this locus. The location of the Pastor QTL was problematic as the DArT markers flanking this QTL were present on very few maps; however, *wPt*-*4572* was located in the same linkage group as *Xmwg634* in an Opata/synthetic map and it is assumed that the Pastor QTL falls in this region. The oligoculm QTL (Suenaga et al. 2003) appears to be distinct from the *Yr46* locus.

## Group 5

**Fig. 5:**
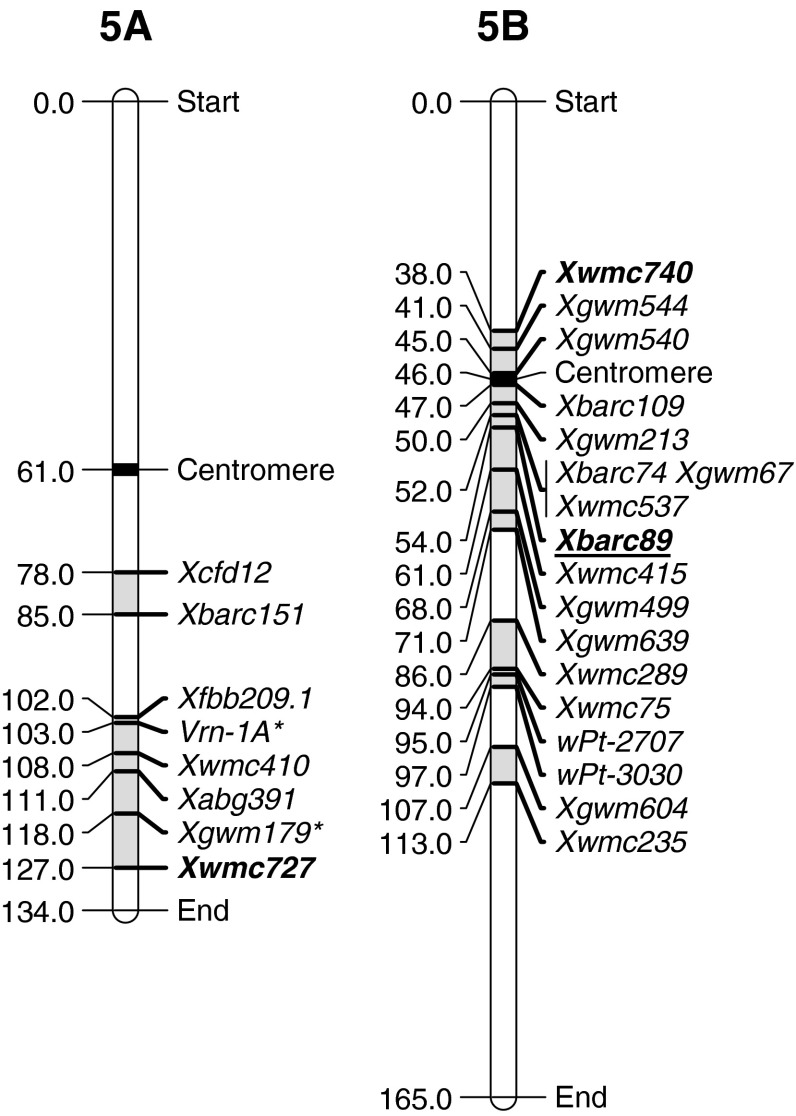
Location of flanking markers associated with stripe rust QTLs on group 5 chromosomes. Marker locations are taken from consensus maps from the CMap website (http://ccg.murdoch.edu.au/cmap/ccg-live/). Map distances for markers in plain text are from “Consensus Maps 2010-11”, underlined markers have map positions estimated from “Consensus 2003” and markers in bold have map positions estimated from “Sommers 2004” consensus maps. *Identified markers that were used as associated (linked) substitutes for QTL markers that were not available on any consensus maps

Group 5 chromosomes have only had QTLs identified on the A and B genomes (Table 5). The 5AL region likely contains two regions associated with resistance. The first was identified in the diploid *Triticum boeticum* and displays seedling susceptibility. LOD and PEV values and the effectiveness in all environments indicate this could be a major gene similar to the HTAP types of resistance. The QRYr5A.2 chromosomal region (Fig. 5) contained QTLs from four parents, Pastor (Rosewarne et al. 2012), Pingyuan 50 (Lan et al. 2010), SHA3/CBRD (Ren et al. 2012a) and Opata 85 (Boukhatem et al. 2002). The first three QTLs were identified in field-based studies and had low to moderate LOD and PEV scores, as well as being inconsistently identified across multiple environments. The Opata 85 QTL was observed in seedling tests; however, it had a low LOD score (suggested but not significant) which fits well with the other loci identified in this region in field-based scores. Hence, all named parents may contain the same locus. Two of the studies (Ren et al. 2012a; Rosewarne et al. 2012) identified QTLs with DArT markers that were not present in any consensus maps. However, *wPt*-*5231*, *wPt*-*1903* and *wPt*-*3334* had previously been associated within 2.9 cM of each other and within 5.2 cM of *Xgwm179* in linkage maps generated using the Cadoux/Reeves population (Francki et al. 2009). In a Berkut/Krichauff population, Huynh et al. (2008) also identified *wPt*-*5231* within 2 cM of the *Vrn*-*A1* locus, which also falls within this genomic region. This is interesting as later maturing lines in a segregating population are often scored lower for stripe rust as the younger leaves look greener. As maturity scores were not disclosed in any of the above populations, it cannot be ruled out that the QTL observed in this region (QRYr5A.2) are related to maturity rather than rust resistance *per se*.

**Table 5:** Summary of stripe rust regions on group 5 chromosomes associated with stripe rust resistance QTLs

Chromosome region	Source	Markers	Field			Infection type			References
			LOD	PEV	Freq	LOD	PEV	Freq	
QRYr5A.1	*T. boeticum*	*Xbarc151* *Xcfd12*	19–21	17–19	3/3	21–23	18–20	3/3^c^	Chhuneja et al. (2008)
QRYr5A.2	Opata 85	*Xfbb209* *Xabg391*				2.8	15	1/1^c^	Boukhatem et al. (2002)
QRYr5A.2	Pastor	*wPt-5231* *wPt-0837 (Xgwm179, Vrn–1A)*	13.2	4–7	2/4				Rosewarne et al. (2012)
QRYr5A.2	Pingyuan 50	*Xwmc410* *Xbarc261*	1.7–5.6	5–20	3/5				Lan et al. (2010)
QRYr5A.2	SHA3/CBRD	*wPt-1903* *wPt-3334, Xwmc727*	2.1–2.4	3–4	0/3				Ren et al. (2012a)
QRYr5B.1	AGS2000	*Xgdm152* *Xwmc740*	3.3	5	1/3				Hao et al. (2011)
QRYr5B.1	Chapio	*Xbarc267* *Xbarc74*	3.6–10.3	6–16	4/8				Yang et al. (2013)
QRYr5B.1	Flinor	*Xgwm67* *Xbarc89*				4.4	37	1/1^b^	Feng et al. (2011)
QRYr5B.1	Libellula	*Xwmc415* *Xwmc537*	4–9	2–10	¾				Lu et al. (2009)
QRYr5B.1	Strampelli	*Xwmc415* *Xwmc537*	3–4.3	3–6	2/3				Lu et al. (2009)
QRYr5B.1	Oligoculm	*Xwmc415*	47.8^a^	3–16	4/4				Suenaga et al. (2003)
QRYr5B.1	Yr16DH70	*wPt-7114* *Xbarc74*	2.8	4.3	1/2	3.5	5.3	2/2^c^	Agenbag et al. (2012)
QRYr5B.1	Camp Remy	*Xgwm639* *Xgwm499, Xgwm544*	6.6–9.7	18–26	4/4				Mallard et al. (2005)
QRYr5B.2	Janz	*wPt-3030* *wPt-2707*	2.5–3.5	6–8	5/6				Bariana et al. (2010)
QRYr5B.2	SHA3/CBRD	*wPt-2707* *Xwmc75*	2.9–5.2	5–8	2/3				Ren et al. (2012a)
QRYr5B.3	Camp Remy	*Xgwm234* *Xgwm604*	9.7–11.2	29–34	4/4				Mallard et al. (2005)
QRYr5B.3	Flinor	*Xwmc235* *Xgwm604*				5	33	1/1^b^	Feng et al. (2011)
QRYr5B.3	Libellula	*Xbarc142* *Xgwm604*	2.5	2.6	¼				Lu et al. (2009)

Markers in parentheses were not described in the original publication but were used as closely associated substitutes on consensus maps where the original markers were not available. Frequency (Freq) describes to number of environments that the locus had a significant QTL, out of the total number of environments tested

^a^Likelihood ratio reported

^b^Infection type scored at high temperatures in glasshouse grown seedlings

^c^Infection type scored in field on adult plants

The 5B chromosome had three clusters of markers associated with stripe rust resistance. Flinor (Feng et al. 2011), Camp Remy (Mallard et al. 2005) and Libellula (Lu et al. 2009) had loci in both QRYr5B.1 and QRYr5B.3. It is likely that these were inherited together. Pedigree information outlined by Feng et al. (2011) showed that these three lines, along with Strampelli, have a common parent in Hatif Inversable, which may have been the donor of these two QTLs. However, Strampelli only contains one of these QTLs (QRYr5B.1). AGS2000 (Hao et al. 2011), Chapio (Yang et al. 2013), Yr16DH70 (Agenbag et al. 2012), Libelulla and Strampelli (Lu et al. 2009) contain QTLs in the QRYr5B.1 cluster of a low to intermediate effect that were inconsistent across environments, raising the possibility that all these lines contain the same gene. Flinor was described to contain a temperature sensitive seedling resistance gene that is more effective in higher temperatures. Yang et al. (2013) showed that the Chapio locus on QRYr5B.1 was only effective in Chinese environments and not in the cooler highland environments in Mexico. If this is the same gene, this difference in effectiveness could be due to environmental factors. Alternately there could be different race virulence in Mexico that renders this gene ineffective. Further studies are needed to determine whether these lines carry the same gene.

The second region on 5B contained QTLs from Janz (Zwart et al. 2010) and SHA3/CBRD (Ren et al. 2012a). These shared a common DArT marker and had similar LOD and PEV values. It seems most likely that these are the same locus if not the same gene.

## Group 6

**Fig. 6:**
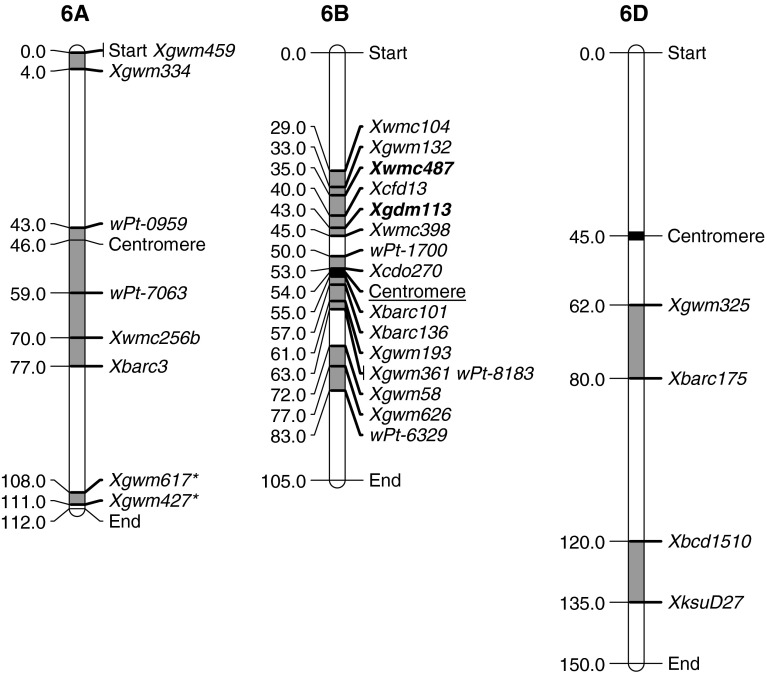
Location of flanking markers associated with stripe rust QTLs on group 6 chromosomes. Marker locations are taken from consensus maps from the CMap website (http://ccg.murdoch.edu.au/cmap/ccg-live/). Map distances for markers in plain text are from “Consensus Maps 2010-11”, underlined markers have map positions estimated from “Consensus 2003” and markers in bold have map positions estimated from “Sommers 2004” consensus maps. *Identified markers that were used as associated (linked) substitutes for QTL markers that were not available on any consensus maps

Chromosome 6A had three clearly defined regions associated with stripe rust resistance and these are likely to be conferred by three distinct genes (Table 6; Fig. 6). The first region is at the telomere of 6AS and was first described by Lin and Chen (2009) as a HTAP gene from the cultivar Express. This locus had high LOD and PEV scores for both final disease severity and lowered IT in field conditions. Hao et al. (2011) identified a locus in Pioneer 26R61 that shared a flanking marker, *Xgwm334*, with the Express locus. Both of these QTLs were effective in all tested environments and these similarities suggest they are associated with the same gene.

**Table 6:** Summary of stripe rust regions on group 6 chromosomes associated with stripe rust resistance QTLs

Chromosome region	Source	Markers	Field			Infection type			References
			LOD	PEV	Freq	LOD	PEV	Freq	
QRYr6A.1	Express	*Xgwm459* *Xgwm334*	5.8–9.8	11–16	3/3	3.6–5.6	8–13	3/3^b^	Lin and Chen (2009)
QRYr6A.1	Pioneer26R61	*Xgwm334* *wPt-7840*	3.1–4.6	6–7	3/3				Hao et al. (2011)
QRYr6A.2	Avocet	*Xbarc3* *xPT-7063*		14	1/1				Lillemo et al. (2008)
QRYr6A.2	Avocet	*Xgwm427* *Xwmc256*	1.9–2.8	6–8	2/3			0/1^b^	William et al. (2006)
QRYr6A.2	Avocet	*wPt-0959*	7.4	2–7	2/4				Rosewarne et al. (2012)
QRYr6A.3	Platte	*378849* *wPt-1642 (Xgwm617, Xcdo836, mwg2053)*	3	6	½				Vazquez et al. (2012)
QRYr6B.1	Bainong 64	*Xwmc487* *Xcfd13*	1.9–2.4	4–6	3/3				Ren et al. (2012b)
QRYr6B.1	Naxos	*Xwmc104* *wPt-0259, wPt-7906*	2.9	6	1/3				Ren et al. (2012a)
QRYr6B.1	Stephens	*Xgwm132* *Xgdm113*	3.5–8.3	30–43	¾				Santra et al. (2008)
QRYr6B.1	Oligoculm	*Xgwm935.1* *Xwmc398*	23^a^	4	2/4				Suenaga et al. (2003)
QRYr6B.2	Janz	*wPt-8183* *wPt-1700*	2.5–2.9	4–8	4/6				Bariana et al. (2010)
QRYr6B.2	Pingyuan 50	*Xgwm361* *Xgwm136*	1.6–2.5	5–8	2/4				Lan et al. (2010)
QRYr6B.2	Recital	*Xcdo270* *Xgwm193*	3.7–3.8	2–4	2/2				Dedryver et al. (2009)
QRYr6B.2	Stephens	*Xbarc101* *Xbarc136*	3.9–6.8	32–45	4/4				Santra et al. (2008)
QRYr6B.2	*T. turgidum* ssp. *dicoccoides* FA15-3	*Xbarc101, Xgwm193*							Uauy et al. (2005)
QRYr6B.3	Pastor	*wPt-6329* *wPt-5176*	5.6	2–4	¾				Rosewarne et al. (2012)
QRYr6B.3	Pavon 76	*Xgwm58* *Xgwm626*	3–6.5	9–19	3/3	2.5	7.9	1/1^b^	William et al. (2006)
QRYr6D.1	Yr16DH70	*Xgwm325* *Xbarc175*	4.2	6.2	1/1			0/1^b^	Agenbag et al. (2012)
QRYr6D.2	W-7984	*Xbcd1510* *XksuD27*				2.4	13.1	1/1^b^	Boukhatem et al. (2002)

Markers in parentheses were not described in the original publication but were used as closely associated substitutes on consensus maps where the original markers were not available. Frequency (Freq) describes to number of environments that the locus had a significant QTL, out of the total number of environments tested

^a^Likelihood ratio reported

^b^Infection type scored in field on adult plants

The second 6A region associated with resistance was close to the centromere but on the long arm of this chromosome. It was identified in three different studies and always derived from the parent Avocet (Lillemo et al. 2008; William et al. 2006; Rosewarne et al. 2012). This QTL had intermediate LOD and PEV scores and although it was not effective in all environments, it is likely to be useful in combination with other genes. Previously we have discussed the presence of minor QTLs in susceptible parents and here we have clear evidence from three different studies that such a QTL is important although its effect in isolation is very minor. The consensus map around the QRYr6A.2 region covers 34 cM, a value that is quite large and does not give confidence in defining this region. This is likely due to the use of some DArT markers in two of the Avocet QTLs and the relative paucity of mapping data surrounding these markers. As better consensus maps of the DArT markers are produced, it is expected that this region would narrow and probably be more focused around the SSR markers identified. Avocet contains the *Thinopyrum elongatum* translocation on 6AL which confers stem rust resistance with *Sr26* (McIntosh et al. 1995). Translocations often carry multiple resistance genes and the positioning of a near-centromeric location for QRYr6A.2 is consistent with it being on the *Sr26* translocation (Rao 1996).

The third region was near the teleomere of 6AL and was identified in Stephens (Vazquez et al. 2012). The closest marker to this QTL was *wPt*-*1642* and although not present on any consensus maps, it had been mapped to this telomeric region in two studies. The marker was mapped to within 4.5 cM of *Xgwm617* in a Cranbrook/Halberd population (Akbari et al. 2006) and to 4.7 cM of *Xgwm427* in a durum population of Colosseo/Lloyd (Mantovani et al. 2008). The close association of these latter two SSR markers and their established position near the telomere of 6AL in consensus maps provides good support for the positioning the stripe rust QTL.

There were three regions of importance in chromosome 6B. The short arm contained QRYr6B.1 which was determined by flanking markers from Bainong 64 (Ren et al. 2012b), Naxos (Ren et al. 2012a), Stephens (Santra et al. 2008) and Oligoculm (Suenaga et al. 2003). Judging by PEV scores, there appears to be two types of loci involved in this region, with the Stephens locus being conferred by a HTAP gene. Stephens also had another HTAP locus in group QRYr6B.2. These were the only two loci in the Santra et al. (2008) study and combined to contribute between 62 and 79 % of variance with both loci having significant effects on IT. The other loci on QRYr6B.1 had much lower PEV and LOD scores and were inconsistently significant across environments indicating the likely involvement of a different gene in this region.

The QRYr6B.2 region contained flanking markers from Janz (Bariana et al. 2010), Pingyuan 50 (Lan et al. 2010), Recital (Dedryver et al. 2009) and the aforementioned Stephens (Santra et al. 2008). Again it seems likely that there are two distinct loci based on the extremely high PEV scores associated with the Stephens QTL, and the relative inconsistency and much lower PEVs of the QTLs associated with the other three lines. This region also contained the HTAP gene *Yr36* (Uauy et al. 2005) derived from *T. turgidum* ssp. *dicoccoides* (Körn.) Thell. accession FA15-3. This gene has been cloned and the predicted protein has similarities to a kinase domain followed by a steroidogenic acute regulatory protein–lipid transfer domain and may play a role in recognizing and binding to lipids in the invading fungus and initiating a programmed cell death signaling cascade (Fu et al. 2009). It seems likely that the Santra et al. (2008) study may have identified the HTAP *Yr36* but it is unknown if the other QTLs in this region contain a similar gene.

The final region on 6B was identified in Pastor (Rosewarne et al. 2012) and Pavon (William et al. 2006) and had moderate to high LOD and PEV scores. The Pavon QTL was also identified with IT data from field studies. These two lines share a common parent, Kalyansona/Bluebird, a line that is known to contribute minor rust resistance genes in CIMMYT germplasm (Rosewarne et al. 2012). From this data it seems likely that a single gene in this region contributes with resistance.

Only two studies have identified resistance loci on chromosome 6D and in both cases on the long arm. Agenbag et al. (2012) identified a minor QTL proximal in 6DL in the line Yr16DH70. Boukhatem et al. (2002) found a seedling resistance gene more distal on 6DL although their QTL analysis stated that this was only suggestive and not significant. These studies were conducted in relatively few environments and more work needs to be done to determine their breeding value.

## Group 7

**Fig. 7:**
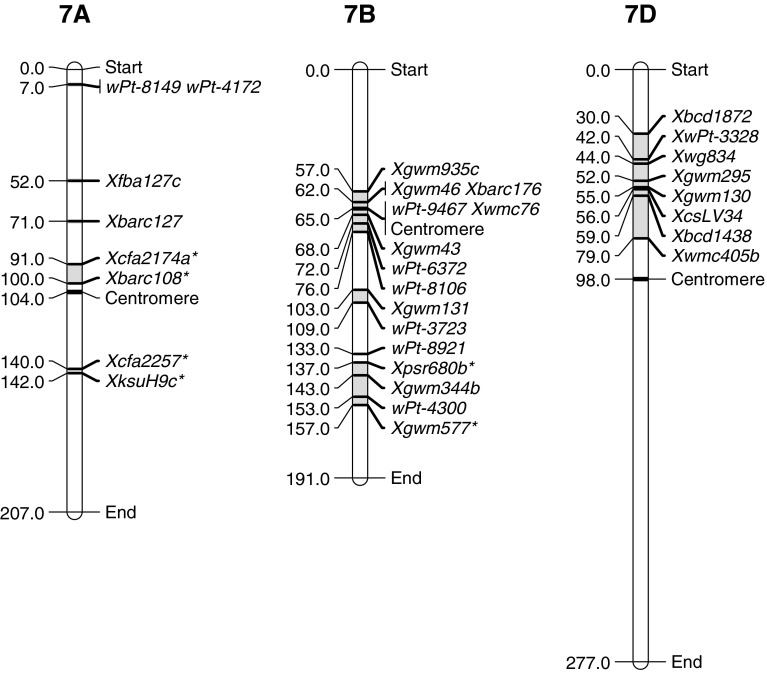
Location of flanking markers associated with stripe rust QTLs on group 7 chromosomes. Marker locations are taken from consensus maps from the CMap website (http://ccg.murdoch.edu.au/cmap/ccg-live/). Map distances for markers in plain text are from “Consensus Maps 2010-11”, underlined markers have map positions estimated from “Consensus 2003” and markers in bold have map positions estimated from “Sommers 2004” consensus maps. *Identified markers that were used as associated (linked) substitutes for QTL markers that were not available on any consensus maps

Chromosome 7A had up to five regions where QTLs were described (Table 7; Fig. 7). All of these were of relative minor effect. Avocet (Rosewarne et al. 2012) had two apparent QTLs, here designated as QRYr7A.1 and QRYr7A.5. The first was located near the telomere of 7AS and the flanking DArT markers had been placed on consensus maps. QRYr7A.2 was derived from Recital and was defined on the consensus map by the marker *Xfba127c* that appears to be centrally located on 7AS (Dedryver et al. 2009). Ren et al. (2012b) identified a fairly minor QTL in a more proximal region of the short arm of 7A (QRYr7A.3) from Jingshuang 16. The CPI133972 (Zwart et al. 2010) QTL in QRYr7A.4 was defined by several DArT markers; however, only *wPt*-*4345* could be found on a linkage map derived from the cross P92201D5-2/P91193D1-10 (Francki et al. 2009) and was flanked by *Xbarc174* (2.2 cM) and *Xcfa2174a* (0.8 cM) tentatively placing this locus near the centromere of 7AS. The second Avocet QTL (Rosewarne et al. 2012) was in a similar location to a QTL identified from Stephens (Vazquez et al. 2012), centrally located on 7AL. Both of these QTLs were described with DArT markers that were not available on consensus maps with the position of the Avocet QTL (*wPt*-*2600*) being inferred by a map generated from a Cadoux/Reeves population (Francki et al. 2009) within 2.8 cM of the SSR marker *Xcfa2257*. The Stephens QTL (wPt-1023) co-segregates with XksuH9c in the Cranbrook/Halberd population (Akbari et al. 2006) and this RFLP marker is well defined on many maps. Both markers defined in the consensus maps are within close proximity to each other. The high LOD scores from the QRYr7A.5 QTLs indicate this as a particularly useful region on chromosome 7AL.

**Table 7:** Summary of stripe rust regions on group 7 chromosomes associated with stripe rust resistance QTLs

Chromosome region	Source	Markers	Field			Infection type			References
			LOD	PEV	Freq	LOD	PEV	Freq	
QRYr7A.1	Avocet	*wPt-8149* *wPt-4172*	5.9	3	2/4				Rosewarne et al. (2012)
QRYr7A.2	Recital	*Xfba127c* *Xbcd129b*	3.8	9	1/1				Dedryver et al. (2009)
QRYr7A.3	Jingshuang16	*Xbarc127*	2.3–2.4	6	2/3				Ren et al. (2012b)
QRYr7A.4	CPI133972	*wPt-4345* *(Xcfa2174, Xbarc108)*	4	11	¼				Zwart et al. (2010)
QRYr7A.5	Avocet	*wPt-2260* *(Xcfa2257)*	9.1	3–6	¾				Rosewarne et al. (2012)
QRYr7A.5	Stephens	*wPt-1023* *(XksuH9c)*	5.2–10	12–20	5/6				Vazquez et al. (2012)
QRYr7B.1	Oligoculm	*Xgwm935.3* *Xgwm46*	30.3^a^	9–17	¾				Suenaga et al. (2003)
QRYr7B.1	Stephens	*wPt-7653* *Xwmc76*	3.1	6	½				Vazquez et al. (2012)
QRYr7B.1	SHA3/CBRD	*Xbarc176* *wPt-8106, wPt-9467*	2.6	8.2	1/3				Ren et al. (2012a)
QRYr7B.2	Alpowa	*Xggp36* *Xgwm131, Xgwm43*	22–29	52–63	4/4	18–25	45–57	3/3^c^	Lin and Chen (2007)
QRYr7B.2	Kukri	*wPt-3723* *wPt-8921*	2.6–3	8–9	6/6				Bariana et al. (2010)
QRYr7B.3	Attila	*Xgwm344*	ND	3.1	3/3				Rosewarne et al. (2008)
QRYr7B.3	Pastor	*wPt-3190* *(Xgwm577, Xpsr680b)*	25.8	4–8	3/3				Rosewarne et al. (2012)
QRYr7B.3	SHA3/CBRD	*Xgwm577* *wPt-4300, wPt-5309*	6.4	14.7	1/3				Ren et al. (2012a)
QRYr7D.1	CD87	*Xwmc405b*	27.4	15	2/2				Bariana et al. (2001)
QRYr7D.1	Chapio	*Xgwm295* *XcsLV34*	6.7–51	10–52	8/8				Yang et al. (2013)
QRYr7D.1	Cook	*Xgwm295*	ND	5–11	3/3				Navabi et al. (2005)
QRYr7D.1	Fukuho-komugi	*Xgwm295*	25.3^a^	11–24	4/4				Suenaga et al. (2003)
QRYr7D.1	Janz	*wPt-3328*	2.5–6.8	7–19	6/6				Bariana et al. (2010)
QRYr7D.1	Janz	*Xwmc405* *wPt-3727, (Xbcd1438)*	4.6–12	15–42	4/4				Zwart et al. (2010)
QRYr7D.1	Kariega	*Xgwm295*	53^a^	29	1/1	14.9^a^	9	1/1^c^	Ramburan et al. (2004)
		*LTN*				22^a^	16	1/1^b^	
QRYr7D.1	Libellula	*XcsLV34*	9.1–15	15–35	4/4				Lu et al. (2009)
QRYr7D.1	Opata 85	*Xwg834* *Xbcd1438*	3.4	13.9	2/2				Boukhatem et al. (2002)
QRYr7D.1	Saar	*wPt-3328* *XcsLV34*	ND	40	1/1				Lillemo et al. (2008)
QRYr7D.1	Strampelli	*XcsLV34*	6.8–17	17–39	5/5				Lu et al. (2009)
QRYr7D.1	Opata 85	*Xwg834*	3–9.7	12–36	3/3	3	12	1/1^c^	Singh et al. (2000b)
		*Xbcd1438, Xbcd1872*				5.4	22	1/1^c^	

Markers in parentheses were not described in the original publication but were used as closely associated substitutes on consensus maps where the original markers were not available. Frequency (Freq) describes to number of environments that the locus had a significant QTL, out of the total number of environments tested. *ND* Not Described

^a^Likelihood ratio reported

^b^Infection type scored at seedling stage in glasshouse

^c^Infection type scored in field on adult plants

Chromosome 7B had three regions associated with resistance. Oligoculm (Suenaga et al. 2003), Stephens (Vazquez et al. 2012) and SHA3/CBRD (Ren et al. 2012a) had QTLs in the centromeric region (QRYr7B.1) and showed intermediate effects against stripe rust. The DArT marker from Stephens (*wPt*-*7653*) was not present on consensus maps and its position was inferred by its close association (0.9 cM) to *Xwmc76* in a Spark/Rialto population (Howes pers. comm.). Region QRYr7B.2, proximal on the long arm of 7B, was identified in Alpowa (Lin and Chen 2007) with a HTAP QTL that had strong effects when lines were scored for final disease severity and IT. They identified two SSR markers that were on the consensus map with *Xgwm131* being within 7 cM of the QTL peak and *Xgwm43* being more than 30 cM from the peak. Kukri (Bariana et al. 2010) also had a QTL in this region identified by the marker *wPt*-*8921*. This QTL was described over a long interval with the aforementioned DArT marker being centrally located, and other markers *wPt*-*6372* and *wPt*-*8106* flanking it by 20.8 and 13 cM respectively. Alpowa and Kukri appear to contain similar loci in regard to the location and effectiveness across all environments. The final region associated with stripe rust on the long arm of 7B was identified in two CIMMYT lines, Attila (Rosewarne et al. 2008) and Pastor (Rosewarne et al. 2012). Both of these loci are linked to a leaf rust QTL by 11–19 cM and as they share a common parent, (Seri M82), they are likely to be the same locus.

The 7D chromosome contains only one region associated with stripe rust resistance and that is the *Yr18* locus. Numerous studies have identified this region (Table 7) on 7DS and it provides strong, stable resistance over every environment tested. Some studies (Ramburan et al. 2004; Singh et al. 2000b) have linked this gene with lowering infection type in the field. These factors would generally suggest a seedling resistance major gene, yet the *Yr18* locus is associated with seedling susceptibility, making it a very important adult plant resistance gene. *Yr18* shares common features with *Yr29* and *Yr46* in that they all confer non-race specific and presumable durable resistance, as well as being effective against multiple pathogens (discussed below).

## Seedling resistance genes

The strongest effect QTLs against rust resistance are generally associated with major, seedling resistance genes. In this review we have summarized several studies where this has been the case. 2AS contains the race-specific seedling resistance gene *Yr17* derived from an *Aegilops ventricosa* translocation (Bariana and McIntosh 1993). This gene was identified in a QTL study by Dedryver et al. (2009) and had high LOD and PEV scores (up to 40 and 43, respectively) which are typical of a major gene. However, this locus was only significant in one of the two environments tested, as the second environment (10 years later) used a rust race that was virulent to *Yr17*. Both Agenbag et al. (2012) and Hao et al. (2011) identified QTLs in this region that had major effects; however, they eliminated the presence of the *Yr17* with the use of molecular markers and by showing that the parents had seedling susceptibility to the rust pathotypes. Chhuneja et al. (2008) also identified an adult plant resistance gene in this region and it was derived from *T. moncoccum*. This gene also had moderate to high effects against the disease (PEV 7-11) that were consistent across all environments tested.

Chromosome 2B also contains a number of seedling resistance genes with *Yr27* from Attila (Rosewarne et al. 2008), *Yr31* from Pastor and Chapio (Rosewarne et al. 2012; Yang et al. 2013) and *Yr7* from Cranbrook (Bariana et al. 2001) having been identified in QTL studies. The seedling resistance gene *Yr31* on 2BS has an intermediate effect on stripe rust infection type and was defeated in Mexico in 2008 with the aforementioned studies showing genotype x environment interactions when the virulent pathotype was used in inoculated field trials. In trials with the avirulent pathotype, PEV scores of between 33–66 % in Pastor and 5–14 % in Chapio were obtained. This locus was significant in all environments in which the avirulent pathotype was used. The QTL associated with *Yr27*, a gene closely linked to *Yr31* with a recombination value of 0.148 (http://www.wheat.pw.usda.gov) was in the Rosewarne et al. (2008) study and was significant in all three environments tested, with PEVs in the range of 4.7–9.3. *Yr27* has been used extensively in germplasm globally. However, virulence to this gene was first identified in South America in the mid-1990s and in South Asia in 2004 and is no longer effective in most parts of the world.

There is virulence for *Yr7* and although this was identified in Cranbrook (Bariana et al. 2001) and is present in Australian germplasm, it has a limited role in controlling stripe rust epidemics.

## High temperature adult plant (HTAP) resistance

Chen and Line (1995) described HTAP resistance as conferred by a class of genes that show susceptibility in seedlings under normal conditions, yet provide generally high levels of resistance in infected adult plants when grown under higher temperatures. There is also evidence of effectiveness at earlier growth stages at higher temperatures with the genes *YrCK* on chromosome 2DS of Cook (Bariana et al. 2001), two loci on chromosome 5B of Flinor (Feng et al. 2009) and in the cloned HTAP gene *Yr36* (Fu et al. 2009). It is assumed that this type of resistance is durable as race-specificity has yet to be shown on any of the characterized loci. Susceptibility under low temperatures is of little consequence as this generally occurs when the wheat plants are young. As plants mature, the temperature usually increases and these loci become effective. Accurate characterization of these loci as HTAP resistance genes is somewhat problematic as specific low and high temperature screens should be completed to show susceptibility; however, most QTL studies only investigate field infections under prevailing temperature regimes, along with seedling tests to confirm susceptibility of parental stocks.

Due to the requirements of a specialized screen to identify HTAP loci, relatively few studies have investigated these loci. Lin and Chen (2007) were the first to localize a HTAP locus when they identified a 7BL locus in “Alpowa” which was designated as *Yr39*. This locus was one of two resistance genes found in “Alpowa” and gave extremely high LOD and PEV scores for both AUDPC and IT data. This highly effective locus was significant across all environments. Santra et al. (2008) identified two HTAP loci on chromosome 6BS from Stephens. Both of these loci were very effective across most environments with AUDPC LOD scores ranging from 3.5 to 8.3 and PEV scores between 30 and 45 %. Lin and Chen (2009) identified two consistent HTAP QTLs from Express on 3BL and 6AS. The 3BL was located near the telomere, indicating it is a unique locus on this chromosome. The 6AS locus was in the same region as a QTL identified by Hao et al. (2011) that had similar LOD and PEV scores, although this later study did not specifically test for HTAP resistance. Both of the QTLs from Express were effective across three environments with high LOD and PEV scores for both AUDPC and IT. A third locus on 1BL was only effective in two of the three environments for AUDPC and did not affect IT. The location of this locus indicates that it is likely different from the well characterized *Yr29* locus.

Carter et al. (2009) and Guo et al. (2008) identified HTAP loci on 2BS from Louise and Luke which group in similar regions along with numerous other resistance loci that include seedling resistances and other small effect QTLs. Guo et al. (2008) identified two loci that shared a common flanking marker and these could possibly belong to a single locus. The loci from Luke and Louise all have high LOD and PEV scores for both AUDPC and IT and were effective in nearly all environments tested. The pedigree data for Luke and Louise were not given, so we are unable to comment on whether they contain the same gene, however, as these were the only HTAP genes identified in these populations, an allelism test would be possible to clarify this issue. Other cultivars that had QTLs in this region include Kariega (Ramburan et al. 2004), Naxos (Ren et al. 2012a), Camp Remy (Boukhatem et al. 2002; Mallard et al. 2005) and Pingyuan 50 (Lan et al. 2010); however, these were not specifically tested for HTAP resistance. Along with the seedling resistance gene *Yr31*, (Rosewarne et al. 2012; Yang et al. 2013), this highlights a very complicated genetic region with multiple resistance loci.

## Pleiotropic adult plant resistances (PAPR)

With the cloning of *Lr34/Yr18/Sr57/Pm38/Ltn1* (Kolmer et al. 2011; Krattinger et al. 2009) came the definitive proof that this very important gene conferred resistance to major biotrophic pathogens of leaf rust, stripe rust, and powdery mildew as well as conferring the phenotypic marker of leaf tip necrosis. Furthermore, QTL mapping indicates that this locus also confers resistance to spot blotch with the *Sb1* designation (Lillemo et al. 2013). Additional studies confirmed its effect on stem rust resistance with the corresponding designation of *Sr57* (RP Singh pers. comm.). There are a number of other loci that are candidates for PAP resistance genes although these need to be confirmed empirically through gene cloning. *Lr46/Yr29/Sr58/Pm39/Ltn2* (Lillemo et al. 2008; Rosewarne et al. 2006) and *Lr67/Yr46/Sr55/Pm46/Ltn3* (Herrera-Fossel et al. 2011; Hiebert et al. 2010) appear to have very similar functional roles in their pleiotropic responses although their absolute values tend to vary across environments.

Evidence of mapping populations containing both *Yr18* and *Yr29* suggest that although these genes generally act additively with most other minor QTLs, they are not completely additive in the presence of each other (Lillemo et al. 2008) and indeed *Yr29* may be less effective against stripe in the presence of *Yr18* (Suenaga et al. 2003; Yang et al. 2013). This suggests that these loci may work on the same molecular pathway in inhibiting fungal growth. Sequence data suggests that *Yr18* is an ABC transporter (Krattinger et al. 2009) and is presumably involved in the export of antifungal compounds out of the cytosol into the apoplast. Despite extensive molecular investigations, Lagudah (2011) has not identified any sequence similarities between the *Yr18* locus and regions surrounding the *Yr29* locus on chromosome 1BL. This suggests a different gene family may be involved in *Yr29* resistance, but this locus somehow taps into the same defensive pathway as *Yr18*. A possible theory of their interaction is that *Yr29* could be a different type of transporter for the same antifungal substrate as *Yr18*, yet may have a higher *K*_*m*_ (Michaelis constant that measures affinity for a particular substrate), resulting in *Yr29* being a less efficient transporter at a given substrate concentration. This is probably the simplest of a number of scenarios that could explain the observed interaction of these two loci.

A fourth candidate for PAP resistance is the *Sr2/Lr27/Yr30/Pbc* locus. This adult plant resistance locus is effective against a variety of pathogens including stem rust, leaf rust, stripe rust and powdery mildew (Mago et al. 2011). Furthermore, a morphological marker of pseudo-black chaff, characterized by a darkening of the glumes and internodes is thought to be pleiotropic or tightly linked to the resistances (Kota et al. 2006). This locus has been effective against stem rust for over 80 years since its original transference to hexaploid wheat from emmer wheat (*T. dicoccum*) by McFadden (1930). The current wide deployment in stem rust prone areas is testament to the durability of *Sr2*. The tight linkage between *Sr2* and *Pbc* was reportedly broken (Mishra et al. 2005), although *Pbc* is known to be a quantitative trait. Again, gene cloning will reveal the true nature of potential pleiotropism of this locus. None-the-less, *Yr30,* associated with *Sr2,* is an important APR locus in wheat germplasm worldwide and has been identified in several QTL studies, generally having a significant effect across the majority of environments tested.

## Other QTLs

The aforementioned classes of resistance genes have relatively few members as identified in QTL studies with the vast majority of QTLs falling into a class that can be characterized as seedling susceptible, not dependent on temperature regimes (as far as known) and are not pleiotropic in nature. These QTLs have quite minor effects as shown by typically low LOD and PEV scores, and are often inconsistently observed across environments. Several studies have identified QTLs being derived from a susceptible parent suggesting that they may be hard to detect when in a genetic background that does not contain other additive resistance loci. However, these loci are critical in the development of durably resistant lines. Placed in combination with HTAP, PAP and other QTLs, they can provide near immunity against stripe rust. Indeed, four to five loci have been shown to confer near immunity (Singh et al. 2000a) and breeding for such resistance, although more complicated than using single, seedling resistance genes, is not overly difficult. This can be greatly enhanced with the improved use of markers or phenotypic selection under severely diseased nurseries.

## Future directions

This review identifies 49 chromosomal regions that contain QTLs that lower stripe rust disease severity and it is likely that many of these regions contain more than one locus. It is expected that with the advent of cheaper genotyping, there will be many more studies identifying regions of importance. Clearly there is an abundance of partial resistance loci that can be used in combating stripe rust epidemics. For the effective deployment of these loci there are still a lot of unanswered questions. Of the loci that appear to be more sensitive to the environment, we need to know in which environments they are effective and what leads to their effectiveness. No doubt there are complicated interactions with prevailing environmental conditions interacting with the timing and severity of an epidemic.

The chromosomal regions identified in numerous studies are likely to contain important loci that are effective across multiple environments and may warrant a greater focus for future research. The PAPR genes feature extensively throughout the research with *Yr18*, *Yr29* and *Yr30* being identified independently in 12, nine and ten studies respectively. Regions QRYr2A.1 and QRYr2B.2 were both identified in seven studies and appear to be gene rich regions containing several seedling resistances and well as some minor QTLs. The QRYr2A.1 also corresponds to a region where several translocations have been incorporated. QRYr2D.2 is a region that contains one of the first identified adult plant resistance genes for stripe rust, *Yr16* with six QTL studies showing this region to have a moderate effect. The regions QRYr4B.1 and QRYr5B.1, identified in seven and eight parents respectively, also tended to show moderate effects that were not identified in all environments tested. These results highlight the importance of these minor QTLs and even though they may not have a significant effect in all environments, these regions do contribute to stable resistance when combined with other loci.

It would also be beneficial to understand how different genetic combinations of the most influential loci interact, such that marker assisted breeding packages can be developed for breeders and ensure diverse deployment of resistance loci is achieved to further enhance durability. A reductionist approach would be to obtain gene designations through the development of single gene lines. Such work was carried out for the leaf rust QTL now designated as *Lr68* (Herrera-Foessel et al. 2012). This approach was to identify lines with a single locus from within a mapping population based on the presence/absence of appropriate QTL flanking markers along with intermediate rust scores. The single gene lines were used as parents in a cross with a susceptible parent and rust scores showed that an intermediate final disease severity score was inherited as a single gene. Problems can arise when isolating some of the minor effect QTLs in that it may be difficult under certain environmental conditions to determine the intermediate resistance phenotype, and that these loci in isolation have a very minor effect that is difficult to detect.

Despite these difficulties, single gene populations do provide the opportunity to dissect the role of these loci as well as aid in marker development and ultimate cloning of the resistance gene. The pyramiding of two or more loci will also be facilitated through the use of closely linked markers and further questions can be answered surrounding genetic interactions and effective gene combinations.

## Abbreviations

AFLP: Amplified fragment length polymorphism
AUDPC: Area under disease progress curve
CAPS: Cleaved amplified polymorphic sequences
cM: Centimorgans
DArT: Diversity array technology
EST: Expressed sequence tag
HTAP: High temperature adult plant
IT: Infection type
LOD: Logarithm (base 10) of Odds
PAPR: Pleiotropic adult plant resistance
PCR: Polymerase chain reaction
PEV: Phenotypic explained variance
QTL: Quantitative trait locus
QTLs: Quantitative trait loci
RFLP: Restriction fragment length polymorphism
RGAP: Resistance gene analogue polymorphism
RAPD: Random amplified polymorphic DNA
SNP: Single nucleotide polymorphism
STS: Sequence-tagged site
